# Effects of the Novel Triazole Fungicide Ipfentrifluconazole on Different Endpoints in Zebrafish Larvae

**DOI:** 10.3390/toxics13100830

**Published:** 2025-09-29

**Authors:** Mingfei Xu, Yilin Huang, Mingrong Qian, Yuanxiang Jin, Hu Zhang

**Affiliations:** 1 Institute of Agro-Product Safety and Nutrition, Zhejiang Academy of Agricultural Sciences, Hangzhou 310021, China; 2 College of Biotechnology and Bioengineering, Zhejiang University of Technology, Hangzhou 310014, China; 3 Key Laboratory of Pollution Exposure and Health Intervention of Zhejiang Province, Interdisciplinary Research Academy, Zhejiang Shuren University, Hangzhou 310015, China

**Keywords:** Ipfentrifluconazole, transcriptomics, zebrafish larvae

## Abstract

The potential hazards of triazole fungicides to non-target organisms necessitate environmental risk assessment. This study, therefore, focused on characterizing the differential toxicity of the enantiomers of Ipfentrifluconazole (IFZ), a new triazole fungicide, in zebrafish larvae using a multi-endpoint approach. Acute toxicity tests determined the LC_50_ values of 1.709 mg/L for rac-IFZ, 1.531 mg/L for (+)-IFZ, and 1.809 mg/L for (−)-IFZ, indicating a higher toxicity of the (+)-enantiomer. To avoid overt mortality while revealing organ-level effects, we chose a concentration of approximately 20% of the LC50 of (+)-IFZ, which is 340 μg/L, as the exposure concentration. Exposure to IFZ induced developmental defects, including swim bladder malformation, cardiac blood pooling, and metabolic disturbances during the early developmental stage of zebrafish. Additionally, cardiac and hepatic development and function were disrupted in zebrafish larvae following IFZ exposure. Biochemical and transcriptomic analyses revealed distinct toxic mechanisms: (+)-IFZ primarily disrupted lipid metabolism through alterations in PPAR signaling pathway and fatty acid degradation, while (−)-IFZ significantly impaired cardiac function by affecting adrenergic signaling in cardiomyocytes and cardiac muscle contraction. Rac-IFZ mainly influenced drug metabolism, particularly cytochrome P450-related pathways. These findings demonstrated the toxic effects of IFZ, emphasizing the need for evaluating environmental and health risks of chiral pesticides. The study provides valuable insights into the molecular mechanisms underlying IFZ toxicity.

## 1. Introduction

Triazole fungicides represent a significant category of agrochemicals extensively employed in farming to manage diverse fungal infections. Owing to their high efficacy, broad-spectrum activity and low toxicity, triazole fungicides are vital in modern agriculture [1]. Triazole fungicides usually have a triazole ring structure, which gives them unique fungicidal activity. This class of fungicides mainly interferes with the biosynthesis of fungal cell membranes by inhibiting sterol demethylase, thereby inhibiting the growth and reproduction of fungi [2]. Since the advent of the first triazole fungicide, triadimefon, in the 1970s, triazole fungicides have experienced rapid development. Subsequently, a series of new triazole fungicides with high efficiency and low toxicity have been developed, such as Flusilazole, Tebuconazole, Epoxiconazole, etc. [3]. The extensive application of triazole fungicides has led to rising awareness of their ecological consequences, particularly regarding non-target organisms like zebrafish. Studies have shown that triazole fungicides can cause adverse effects in zebrafish, manifested as abnormal behavior, decreased swimming ability [4], and even death [5]. Triazole fungicides can induce oxidative stress in zebrafish, leading to cell and DNA damage. Some triazole fungicides can act as endocrine disruptors in zebrafish, altering their hormonal balance and consequently impairing reproductive system function [6].

Ipfentrifluconazole (IFZ) is a new triazole fungicide developed by BASF in recent years. It contains an asymmetric chiral carbon atom and is composed of a pair of enantiomers. IFZ functions by disrupting ergosterol production in pathogenic organisms, compromising cellular membrane integrity. This interference impedes critical fungal structures including attachment cells, haustoria, hyphal growth, and spore formation. However, investigations into IFZ’s ecological consequences for aquatic systems and non-target species remain notably limited. However, mefentrifluconazole (MFZ), which only differs from IFZ in its chemical structure by methyl and isopropyl, has been widely studied by researchers. Studies have shown that MFZ exposure can cause *Eisenia fetida* cell membrane damage and apoptosis, and the selectivity of apoptosis may be dominated by the mitochondrial pathway. Comparative studies reveal distinct bioactivity between the MFZ stereoisomers. The S-(+)-enantiomer demonstrates specific upregulation of mitochondrial structural components, resulting in exacerbated mitochondrial fragmentation. More significantly, this isomer potently disrupts bioenergetic processes through dual inhibition of mitochondrial respiratory chain complexes I (NADH dehydrogenase) and IV (cytochrome c oxidase), thereby impairing electron flux and ATP generation capacity to a greater extent than its R-(−)-counterpart [7]. In addition, experimental evidence demonstrates that MFZ exposure induces enantioselective developmental toxicity in zebrafish across multiple organ systems. Cardiac manifestations include embryonal bradycardia, pericardial and yolk sac deformities, and significant downregulation of cardiac-specific genetic markers. Concurrently, neurodevelopmental impacts are observed through suppressed spontaneous movements, altered locomotor activity such as swimming speed and distance, and modulated expression of neurotoxicity-related genes. These effects consistently show stereochemical dependence, with enantiomers exhibiting enhanced bioactivity [8]. In addition, MFZ also causes enantioselective changes in the metabolomics of adult zebrafish livers [9], and MFZ exposure impairs liver structure and liver lipid metabolism in mice, impairs intestinal barrier function, and promotes inflammation [10]. Therefore, it is of significance to explore whether IFZ has similar toxic effects to MFZ or whether it has unique toxic effects.

The zebrafish (*Danio rerio*) has become a widely adopted and instrumental model organism for toxicological research, particularly for assessing the safety of pesticides, environmental contaminants, and pharmaceuticals. With approximately 70% genetic homology to humans and conserved core physiological pathways, zebrafish exhibit remarkable similarities to mammalian systems. Zebrafish exhibit significant structural and functional conservation in their hepatic, renal, and cardiovascular systems when compared to mammals. This high degree of homology establishes the zebrafish as a powerful and versatile model organism for studying the mechanisms of chemical toxicity and for evaluating the physiological effects of toxicants [11]. Zebrafish embryos and larvae are transparent in the early stages of development, and processes such as organ formation, blood circulation, and cell apoptosis can be directly observed under a microscope. This feature makes it an ideal model for studying developmental toxicity, teratogenicity, and cardiotoxicity [12]. In addition, zebrafish have the characteristics of strong reproductive capacity and rapid embryonic development, which is suitable for high-throughput toxicity screening [13].

This study first explored the effects of IFZ on the growth and development of zebrafish embryos. Then, we explored the effects of IFZ on the development and function of the heart and liver of zebrafish larvae. Finally, the transcriptomic analysis was used to analyze the selective effects of IFZ racemates and enantiomers. At present, there is a lack of research on the effects of IFZ on the growth and development of zebrafish and the functions of various organs. This study will provide some theoretical basis for the toxicological effects of IFZ.

## 2. Materials and Methods

### 2.1. Chemicals

Ipfentrifluconazole (IFZ) (CAS No:1417782-08-1) was purchased from Altascientific (Tianjin, China). The enantiomers were separated according to the research method reported by Li et al. [14]. IFZ was dissolved in dimethyl sulfoxide (DMSO) and subsequently stored at −20 °C to maintain stability until required for experiments.

### 2.2. Zebrafish Breeding and Embryo Collection

This study employed wild-type zebrafish alongside two transgenic models, *Tg(myl7:EGFP)* and *Tg(-1.7apoa2:GFP)*, which were obtained from the China Zebrafish Resource Center in Wuhan. All zebrafish were housed in glass aquarium systems supplied with activated carbon-filtered and dechlorinated tap water, maintained at 28 ± 1 °C. The facility maintained a 14 h light/10 h dark photoperiod to regulate circadian rhythms. For nutritional requirements, the zebrafish received feedings of freshly hatched brine shrimp twice each day. Breeding protocols involved placing adult zebrafish in specialized mating tanks overnight at a ratio of two females to one male. The following morning, spawning was induced through partition removal between the sexes, with subsequent embryo collection completed within a 30 min window followed by rinsing to prepare for experimental procedures.

### 2.3. Detection of Acute Toxicity

The acute toxicity of IFZ to zebrafish embryos was evaluated according to OECD Test Guideline No. 236. The exposure period spanned from 2 h post-fertilization (hpf) to 96 hpf. In preliminary tests, six IFZ concentrations (400, 800, 1200, 1600, 2000, and 2400 μg/L) were examined. For each concentration, 30 fertilized embryos were transferred into six-well plates containing 5 mL of exposure solution, with three replicates per group. The exposure solution was renewed every 24 h. During the experimental period, culture plates were covered to minimize solvent evaporation and maintained in an incubator at a constant 28 °C under a 14/10 h light/dark photoperiod. All tests were initiated at 2 h post-fertilization (2 hpf) using non-dechorionated embryos. Daily observations included recording hatching success and mortality rates. For data analysis, IFZ concentrations were log10-transformed and plotted on the x-axis, while mortality rates were represented on the y-axis. A dose–response curve was generated using nonlinear regression to estimate the median lethal concentration (LC_50_).

Based on the LC_50_ values of IFZ obtained from the experiments above, an exposure concentration of 340 μg/L was chosen. Stock solutions of both the racemate and the separate enantiomers were prepared and then diluted using E3 medium to reach the target concentration of 340 µg/L. Four experimental groups were established: Control (CON), rac-IFZ (rac), (+)-IFZ (+), and (−)-IFZ (−). The CON group served as the DMSO solvent control, with DMSO levels in all test solutions kept at or below 0.1% (*v*/*v*). For each experimental condition, a total of 120 embryos were carefully transferred into individual glass beakers, each containing 200 mL of the designated test solution. The larvae were maintained under a constant temperature of 28 °C and a photoperiod of 14 h light and 10 h darkness. Every 24 h, three-quarters of the test medium was refreshed. Following 7 days of continuous exposure, zebrafish larvae were gathered for further analysis.

### 2.4. Biochemical Indicators Analysis

The tissue samples were first homogenized in phosphate-buffered saline (PBS) and subsequently subjected to centrifugation at 4000 rpm for a duration of 10 min under 4 °C to isolate the supernatant. A precise 20 μL aliquot of the resulting clear upper phase was then carefully collected from each processed sample. Total protein content was quantified using a BCA Protein Assay Kit (Sangon, Shanghai, China). Furthermore, key metabolic biomarkers, including triglyceride, total cholesterol, glucose, and pyruvate, were assessed employing commercial enzymatic assay kits (Nanjing Jiancheng Bioengineering Institute, Nanjing, China) in strict accordance with the manufacturer’s protocols.

### 2.5. RNA Extraction and Transcriptome Analysis

Total RNA isolation was performed with TRIZOL reagent (Vazyme, Nanjing, China), and RNA integrity was assessed using the RNA Nano 6000 Assay Kit on an Agilent Bioanalyzer 2100 system (Agilent Technologies, Santa Clara, CA, USA). Sequencing libraries were prepared with the NEBNext^®^ Ultra™ RNA Library Prep Kit (NEB, Ipswich, MA, USA), and their quality was evaluated via the Qubit^®^ 2.0 Fluorometer (Thermo Fisher Scientific, Waltham, MA, USA) as well as quantitative real-time PCR (qRT-PCR). Cluster generation was carried out on the Illumina cBot system (Illumina, Inc., San Diego, CA, USA) with the TruSeq PE Cluster Kit v3-cBot-HS (Illumina, Inc., San Diego, CA, USA), and the libraries were subsequently sequenced on the Illumina NovaSeq platform (Illumina, Inc., San Diego, CA, USA) to generate 150 bp paired-end reads. Raw sequencing data were preprocessed by eliminating adapter sequences, low-quality reads, and poly-N segments using in-house Perl scripts. Differential gene expression analysis was performed using DESeq2 version 1.20.0, with differentially expressed genes identified based on an adjusted *p*-value cutoff of less than 0.05 after the Benjamini–Hochberg correction. Significant genes were functionally annotated using Gene Set Enrichment Analysis (GSEA) and KEGG pathway analysis, both performed with the clusterProfiler package in R 4.3.1.

### 2.6. Gene Expression Analysis

All experimental conditions were analyzed with six independent biological replicates, with each replicate consisting of 30 zebrafish larvae. Total RNA was isolated with TRIzol reagent (Vazyme, Nanjing, China), and complementary DNA (cDNA) was synthesized through reverse transcription. Quantitative real-time PCR (qPCR) was subsequently carried out using a SYBR Green master mix (Vazyme, Nanjing, China) on a Bio-Rad CFX Connect Real-Time PCR Detection System. The *ef1α* gene was used as an endogenous control for data normalization, and the relative expression levels of the genes of interest were determined based on the comparative threshold cycle (2^−ΔΔCT^) method. The loop conditions and relative gene expression follow the previously established protocol [15,16].

### 2.7. Observation of Heart Morphology of Tg(myl7: EGFP)

To assess cardiac development, embryos were distributed into six-well plates with 30 embryos per well. The embryos were continuously exposed for 96 h. Afterwards, 10 randomly selected larvae were observed under a fluorescence microscope for cardiac morphology and related cardiac parameters were measured. Zebrafish were photographed at the end of ventricular systole and at the end of ventricular diastole, and the software ImageJ-win32 was used to quantify the ventricular areas at the end of systole and diastole. The formulas for calculating stroke volume and ejection fraction were as follows:

stroke volume = VEDA − VESA

ejection fraction = (stroke volume)/VEDA

fractional shortening = (Dd − Ds)/Dd

VEDA: Ventricular end-diastolic area.

VESA: Ventricular end-systolic area.

Dd: Ventricular end-diastolic diameter.

Ds: Ventricular end-systolic diameter.

### 2.8. Observation of Liver Morphology of Tg(-1.7apoa2: GFP)

Ten larval zebrafish (96 hpf) were randomly selected to observe the liver development. Thirty embryos were placed in each well of a six-well plate, and after 7 consecutive days of exposure, the liver morphology was observed under a microscope and the liver area and fluorescence intensity of the liver region were measured.

### 2.9. Statistical Analysis

All statistical analyses were conducted using GraphPad Prism version 8.0. Results were presented as mean ± SEM. Group comparisons were carried out by one-way ANOVA, with asterisks (*) used to mark significant differences compared to the control group (* *p* < 0.05, ** *p* < 0.01), and hashtags (#) indicating significant differences between experimental treatments (# *p* < 0.05).

## 3. Results

### 3.1. Developmental Toxicity of IFZ to Zebrafish Larvae

**Figure 1:**
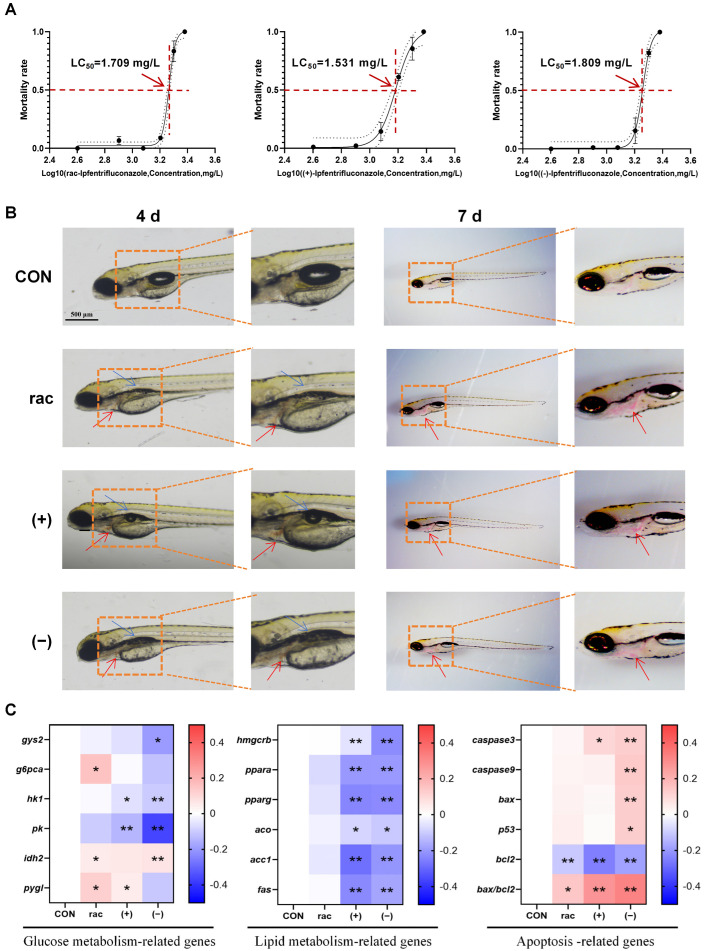
Developmental toxicity of IFZ to zebrafish larvae. (**A**) Semi-mortality curve of zebrafish larvae exposed to different concentrations at 4 d. (**B**) Morphological images of larval zebrafish at 4 d and 7 d. The blue arrows indicate abnormal swim bladder development while the red arrows indicate blood pooling in the pericardial area. (**C**) The mRNA levels of genes related to glucose and lipid metabolism and apoptosis (*n* = 6). Data are presented as the mean ± SEM. Asterisks indicate significant difference between treatment group and control (* *p* < 0.05; ** *p* < 0.01).

This study evaluated the acute toxic effects of IFZ on zebrafish larvae following a 96 h exposure period across a gradient of concentrations, during which mortality was systematically documented. The calculated LC_50_ values were 1.709 mg/L for rac-IFZ, 1.531 mg/L for (+)-IFZ, and 1.809 mg/L for (−)-IFZ (Figure 1A). Subsequent morphological observations on days 4 and 7 revealed incomplete swim bladder development in IFZ exposure larvae, along with evident blood pooling in the cardiac region. These findings suggested potential impairment of heart development or possible thrombotic events (Figure 1B). Next, the TC, TG, glucose, and pyruvate levels were measured. The results indicated a significant increase in triglyceride (TG) content following IFZ exposure. The TC and glucose contents in the (−)-IFZ group also increased significantly (Table 1). Moreover, the expression levels of mRNA linked to glucose and lipid metabolism, along with apoptosis-related genes, were quantitatively assessed. The findings revealed that IFZ exposure disrupted glucose metabolism and triggered apoptosis in the larvae, with the (−)-IFZ isomer exerting a more pronounced effect. Both (+)-IFZ and (−)-IFZ treatments affected the lipid metabolism of zebrafish larvae (Figure 1C).

### 3.2. Cardiotoxicity of IFZ to Zebrafish Larvae

**Figure 2:**
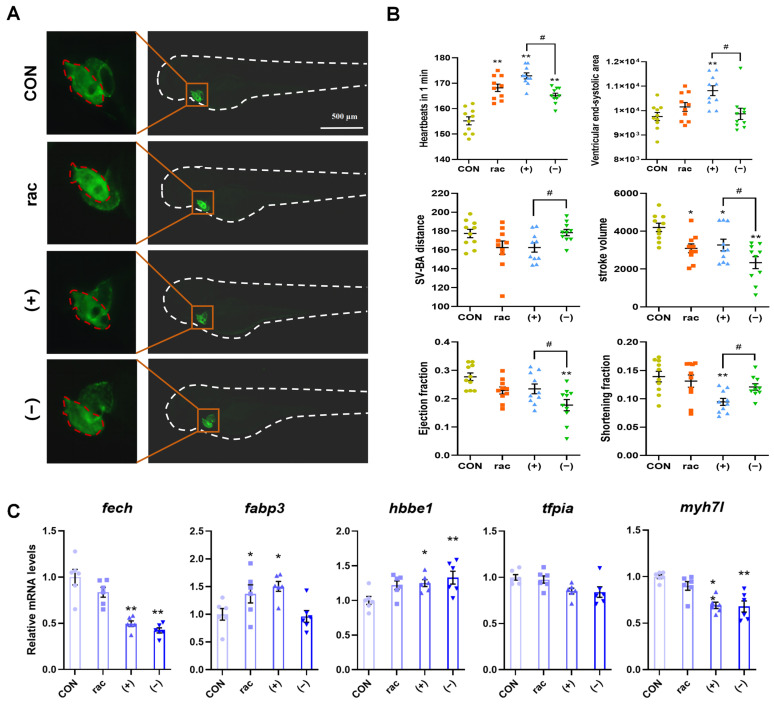
Cardiotoxicity of IFZ to zebrafish larvae. (**A**) Heart morphology of *Tg(myl7: EGFP)* transgenic zebrafish. (**B**) Determination of heart-related indicators (*n* = 10). (**C**) mRNA levels of cardiac development and function-related genes (*n* = 6). Data are presented as the mean ± SEM. Asterisks indicate significant difference between treatment group and control (* *p* < 0.05; ** *p* < 0.01). Hashtags indicate significant difference between different treatment groups (# *p* < 0.05).

Since obvious abnormalities were found in the heart area of the larvae in the IFZ treatment group when observing the morphology of the larvae on the 4th and 7th days, the transgenic larvae *Tg(myl7: EGFP)* were used to observe the heart morphology and measure heart-related indicators. As demonstrated in Figure 2A,B, IFZ exposure significantly elevated larval heart rate while reducing stroke volume. Notably, (+)-IFZ treatment additionally increased ventricular end-systolic area and decreased shortening fraction. (−)-IFZ exposure significantly reduced larval ejection fraction. Subsequent analysis of cardiovascular-related gene expression revealed significant alterations in mRNA levels for both (+)-IFZ and (−)-IFZ treatment groups (Figure 2C).

### 3.3. Hepatotoxicity of IFZ to Zebrafish Larvae

**Figure 3:**
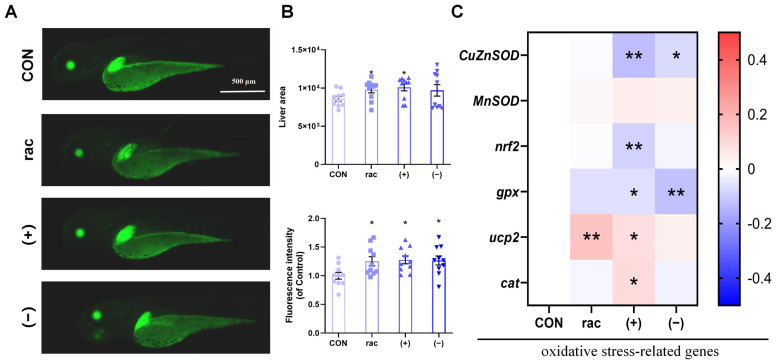
Hepatotoxicity of IFZ to zebrafish larvae. (**A**) Liver morphology of *Tg(-1.7apoa2: GFP)* transgenic zebrafish. (**B**) Quantification of larval liver area and fluorescence intensity (*n* = 10). (**C**) The mRNA levels of oxidative stress-related genes (*n* = 6). Data are presented as the mean ± SEM. Asterisks indicate significant difference between treatment group and control (* *p* < 0.05; ** *p* < 0.01).

Building upon the observed lipid metabolic disruptions, we employed *Tg(-1.7apoa2:GFP)* zebrafish to assess hepatotoxicity (Figure 3A). IFZ exposure universally increased hepatic area and GFP fluorescence intensity, with rac-IFZ and (+)-IFZ showing particularly pronounced hepatomegaly (Figure 3B). Molecular analysis revealed enantiomer-specific oxidative stress responses, where (+)-IFZ induced the most substantial alterations in oxidative stress-related gene expression profiles (Figure 3C).

### 3.4. Transcriptomic Analysis of IFZ Exposed to Larval Zebrafish

**Figure 4:**
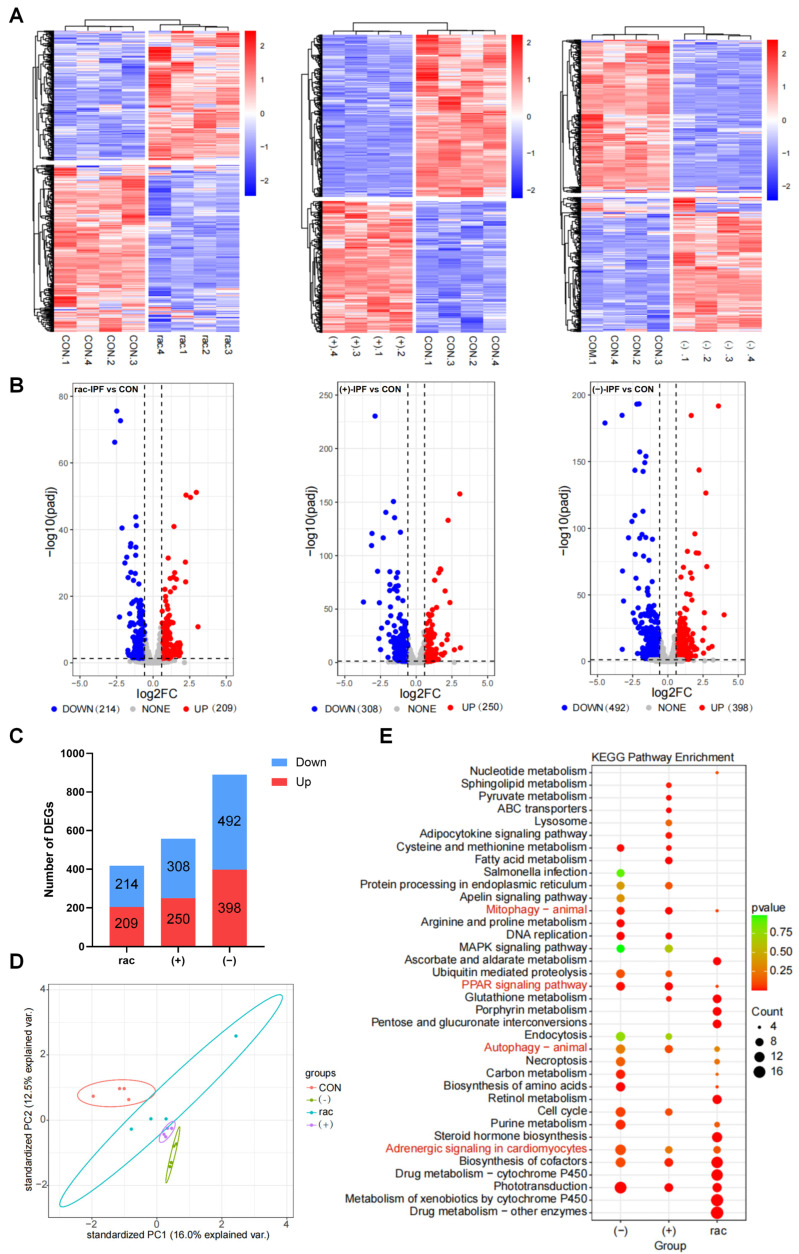
Transcriptomic analysis of IFZ exposed to larval zebrafish. (**A**) Heatmap of cluster analysis using fpkm of differentially expressed genes (DEGs). (**B**) Volcano map based on DEGs (*p* < 0.05 and fold change > 1.5). (**C**) The number of DEGs in each treatment group. (**D**) Principal component analysis (PCA). (**E**) Enrichment analysis of KEGG pathway map based on all DEGs.

To elucidate the molecular mechanisms underlying IFZ toxicity, we conducted transcriptome profiling of exposed zebrafish. Quality control assessment demonstrated strong reproducibility, as evidenced by hierarchical clustering where biological replicates from each treatment group formed distinct clusters in the heatmap analysis. This consistent grouping pattern confirmed the reliability of our transcriptional data for subsequent differential expression analysis (Figure 4A). The results in Figure 4B,C showed that the rac-IFZ treatment group had 423 differentially expressed genes (DEGs), the (+)-IFZ treatment group had 558 DEGs, while the (−)-IFZ treatment group had 890 DEGs and had the largest number of up-regulated and down-regulated genes (Figure 4B,C). Principal component analysis revealed that all treatment groups were distinctly clustered and separated from the control cohort. Notably, the (−)-IFZ group was the most removed from the control, underscoring its pronounced differential effect (Figure 4D). Subsequent KEGG pathway enrichment analysis of these DEGs identified significant involvement in processes including drug metabolism, cardiac development, lipid metabolism, apoptosis, and autophagy (Figure 4E).

### 3.5. Comparison of Transcriptomes Between Racemates and Enantiomers of IFZ

**Figure 5:**
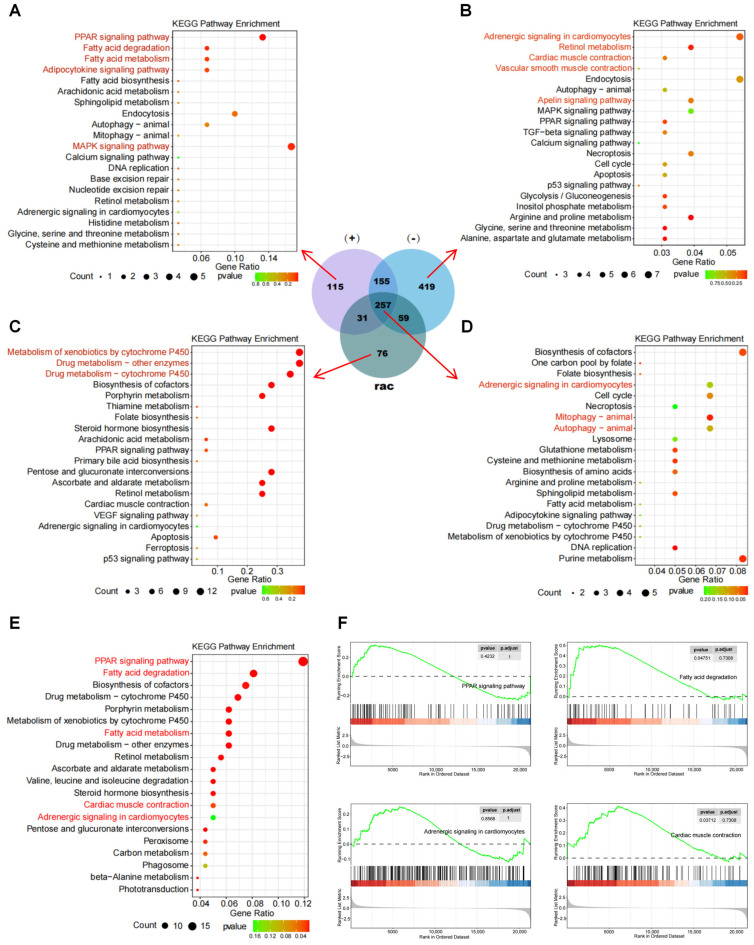
Comparison of transcriptomes between racemates and enantiomers of IFZ. Enrichment analysis of KEGG pathway map based on DEGs unique to the (+)-IFZ group (**A**), (−)-IFZ group (**B**), and rac-IFZ group (**C**). (**D**) Enrichment analysis of KEGG pathway map based on common DEGs across all three treatment groups. (**E**) Enrichment analysis of KEGG pathway map based on DEGs between (+)-IFZ and (−)-IFZ groups. (**F**) GSEA analysis of PPAR signaling pathway, fatty acid degradation, adrenergic signaling in cardiomyocytes, and cardiac muscle contraction.

To further explore the differences between racemates and enantiomers, the DEGs in each group were compared using a Venn diagram, and then the DEGs specific to each treatment group were subjected to KEGG enrichment analysis. Consistent with the volcano plot analysis, the (−)-IFZ treatment group exhibited the highest number of unique DEGs. Functional enrichment analysis revealed these DEGs were predominantly associated with cardiac development and function pathways, including adrenergic signaling in cardiomyocytes, apelin signaling pathway and cardiac muscle contraction (Figure 5B). Transcriptomic profiling revealed significant enrichment of lipid metabolism pathways in (+)-IFZ-exposed zebrafish, with the PPAR signaling pathway showing particularly strong activation. Additional affected metabolic processes included fatty acid degradation and biosynthesis pathways, suggesting disruption of lipid homeostasis (Figure 5A). The rac-IFZ treatment group exhibited the fewest unique DEGs, with pathway enrichment analysis revealing predominant involvement in xenobiotic metabolic processes, particularly cytochrome P450-mediated pathways including metabolism of xenobiotics by cytochrome P450, drug metabolism-cytochrome P450 and other drug-metabolizing enzyme systems (Figure 5C). Additionally, KEGG pathway enrichment analysis was conducted on the DEGs common to all three treatment groups. The findings revealed significant enrichment in key biological pathways, including cardiac adrenergic signaling, autophagy regulation, and mitophagy processes (Figure 5D).

To further investigate the distinctions between IFZ enantiomers, we conducted KEGG enrichment analysis on the DEGs comparing the (+)-IFZ and (−)-IFZ treatment groups, with the (−)-IFZ group serving as the control. This analysis identified significant enrichment in pathways related to lipid metabolism, cardiac development, and cardiac function (Figure 5E). Subsequently, GSEA was performed focusing on the fatty acid degradation, PPAR signaling pathway, adrenergic signaling in cardiomyocytes, and cardiac muscle contraction. The results demonstrated a marked upregulation of these four pathways in the (+)-IFZ group relative to the (−)-IFZ group (Figure 5F).

**Figure 6:**
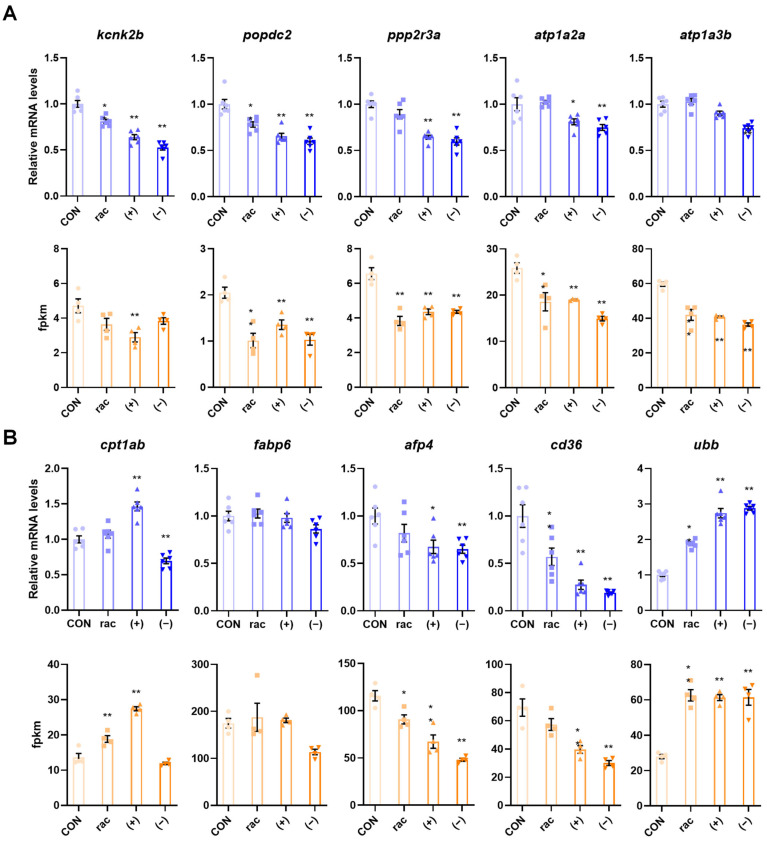
Validation of transcriptomic results. (**A**) Five genes associated with adrenergic signaling in cardiomyocytes were verified using qRT-PCR and verified using the fpkm values in the transcriptome data. (**B**) Five genes associated with PPAR signaling pathway were verified using qRT-PCR (*n* = 6) and verified using the fpkm values (*n* = 4) in the transcriptome data. Data are presented as the mean ± SEM. Asterisks indicate a significant difference between treatment group and control (* *p* < 0.05; ** *p* < 0.01).

To verify the transcriptome results, five DEGs linked to adrenergic signaling in cardiomyocytes and the PPAR signaling pathway were chosen for validation via RT-qPCR. The experimental results demonstrated consistent expression patterns with the transcriptome fpkm values, thereby confirming the reliability of the RNA-seq data (Figure 6).

## 4. Discussion

Triazole fungicides are a class of highly effective and broad-spectrum organic heterocyclic fungicides. They inhibit 14α-demethylation reactions mediated by fungal cytochrome P450 (CYP51), block ergosterol synthesis, and thus destroy cell membrane structures and exert antibacterial effects [17]. Nevertheless, their possible adverse effects on non-target species have raised growing concerns. Research indicates that triazole compounds can disrupt steroid hormone metabolism, potentially causing endocrine dysfunction through inhibition of key mammalian CYP450 enzymes, including CYP19 and CYP17 [18]. Previous studies have identified that several triazole fungicides, including tebuconazole and propiconazole, can induce excessive production of reactive oxygen species (ROS). This oxidative burst leads to cellular oxidative stress and subsequent DNA damage, ultimately contributing to genomic instability. These effects may potentially disrupt embryogenesis and exhibit teratogenic properties [2,19]. Their environmental persistence and bioaccumulation further increase ecotoxicological risks. As a new type of triazole fungicide, IFZ has relatively few studies on its toxicological effects. Therefore, a comprehensive investigation into the molecular toxicological mechanisms of IFZ is essential for assessing its potential risks to environmental safety and human health.

The median lethal concentration (LC_50_) serves as a fundamental indicator for evaluating acute toxicity, specifically measuring the short-term lethal effects of triazole fungicides on zebrafish larvae. This well-established parameter offers critical insights into the immediate toxicological potential of chemical compounds [20]. In this study, LC_50_ determination revealed that (+)-IFZ exhibited marginally greater acute lethality compared to both rac-IFZ and (−)-IFZ. Morphological analysis further demonstrated that IFZ exposure induced significant swim bladder malformations in developing zebrafish larvae. The swim bladder is an important organ of zebrafish larvae, responsible for regulating buoyancy and assisting movement and breathing [21,22]. Abnormal development of the swim bladder, such as inflation failure, morphological deformity, or abnormal size, usually reflects abnormal development and impaired behavior and survival ability [23,24]. At the same time, it was observed that the pericardial area of larvae showed obvious blood pooling after IFZ exposure, suggesting that IFZ exposure could cause abnormal development and impaired function of the heart of zebrafish larvae [25]. Subsequently, the expression of genes involved in glucose metabolism, lipid metabolism, and apoptosis was quantified, along with physiological biomarkers including TG, TC, and glucose levels in the larvae. The results showed that TG levels increased significantly after IFZ exposure, and the expression of key genes for fatty acid β-oxidation, peroxisome proliferator-activated receptor alpha (*ppara*), and acyl-CoA oxidase (*aco*), was significantly downregulated after (+)-IFZ and (−)-IFZ exposure, which promoted lipid accumulation [26,27]. In addition, the expression of lipid synthesis-related genes 3-hydroxy-3-methylglutaryl-CoA reductase b (*hmgcrb*), acetyl-CoA carboxylase 1 (*acc1*), and fatty acid synthase (*fas*) was also significantly inhibited, which might be due to negative feedback regulation triggered by excessive lipid accumulation [28,29,30]. After rac-IFZ exposure, the expression of key enzyme gene glucose-6-phosphatase catalytic subunit a (*g6pca*) in the gluconeogenesis process and the rate-limiting enzyme gene glycogen phosphorylase l (*pygl*) in glycogenolysis was significantly upregulated, indicating that hepatic glucose output increased and glycogen reserves decreased [31]. After (+)-IFZ and (−)-IFZ exposure, the expression of pyruvate kinase (*pk*) and hexokinase 1 (*hk1*), key genes in the glycolysis process, was significantly downregulated, indicating that the glycolysis process was inhibited and glucose utilization was impaired [32]. Finally, the expression levels of apoptosis-related genes were measured and it was found that after IFZ exposure, the expression of the apoptosis-promoting gene bcl-2-associated x (*bax*) was upregulated, and the expression of the apoptosis-inhibiting gene b-cell lymphoma 2 (*bcl2*) was significantly downregulated [33]. The significant increase in the *bax*/*bcl2* ratio indicated that IFZ exposure significantly promoted apoptosis. In summary, IFZ exposure was found to disrupt normal growth and development, impair glucose and lipid homeostasis, and promote apoptosis in zebrafish larvae.

As a model organism, zebrafish is extensively utilized in toxicological studies owing to its favorable characteristics, including embryonic transparency, rapid developmental cycles, and high genetic homology with mammals. Studies have shown that zebrafish embryos are highly sensitive to environmental pollutants, especially in terms of cardiac development, and are often used as an important model for evaluating cardiac toxicity [34,35]. As evident from Figure 1B, pronounced blood accumulation was observed within the pericardial region of the zebrafish larvae. The effect of IFZ on zebrafish cardiac development was investigated using cardiac transgenic zebrafish. IFZ exposure caused significant changes in cardiac-related parameters, and there were significant differences between (+)-IFZ and (−)-IFZ exposure, indicating that IFZ exposure affects zebrafish cardiac development and function. Ferrochelatase (FECH), the terminal enzyme in heme biosynthesis [36], is critical for endothelial cell proliferation in vitro and choroidal neovascularization in vivo [37]. Separately, fatty acid binding protein 3 (FABP3), a low-molecular-weight lipid chaperone, regulates lipid transport, metabolism, and transcriptional control [38,39]. Pathologically elevated FABP3 expression promotes cardiomyocyte apoptosis in myocardial infarct zones and border regions, worsening cardiac dysfunction through impaired left ventricular ejection fraction [40]. Hemoglobin subunit epsilon 1 (*hbbe1*) is a key marker gene related to cardiovascular development [41]. Tissue factor pathway inhibitor alpha (*tfpia*) is a negative regulator of coagulation in zebrafish and silencing it leads to thrombosis [42]. The changes in the above gene expression levels indicated that IFZ exposure could lead to abnormal cardiovascular development and cardiac function damage in zebrafish larvae, especially (+)-IFZ and (−)-IFZ exposure.

The liver plays a pivotal role in regulating lipid homeostasis, functioning as the primary site for multiple metabolic processes including fatty acid synthesis, lipid degradation, lipoprotein assembly and secretion, as well as the storage and mobilization of lipid droplets. It actively coordinates systemic lipid balance through the integration of hormonal and nutritional signals, thereby exerting profound effects on whole-body energy metabolism [43]. Our results indicated that exposure to IFZ led to substantial changes in the expression of genes associated with lipid metabolism (Figure 1C). To further examine IFZ’s hepatic effects, we employed liver-specific transgenic zebrafish models. Quantitative analyses demonstrated that IFZ exposure increased liver size and disrupted antioxidant system homeostasis, with (+)-IFZ exhibiting more pronounced effects. These conclusions were supported by measurements of hepatic area, fluorescence intensity, and oxidative stress-related gene expression profiles in larvae.

Next, transcriptomic analysis was used to further explore the differences in toxic effects between rac-IFZ, (+)-IFZ and (−)-IFZ. The (−)-IFZ group had the largest number of DEGs, and in the PCA analysis result graph, it was the farthest from the CON group, indicating that (−)-IFZ exposure would lead to more significant differences. KEGG enrichment analysis was performed on the DEGs unique to each group. The analysis revealed predominant enrichment of lipid metabolism-associated pathways in the (+)-IFZ group, with the PPAR signaling pathway emerging as a pivotal transcriptional regulatory mechanism. This pathway plays a pivotal role in regulating lipid homeostasis, mediating inflammatory processes, and contributing to cardiovascular pathophysiology [44,45], indicating that (+)-IFZ mainly affects lipid metabolism in larvae. The (−)-IFZ group was mainly enriched in heart-related signaling pathways, among which adrenergic signaling in cardiomyocytes regulates the response of cardiomyocytes to catecholamines such as epinephrine and norepinephrine, activates the Gαs-PKA pathway through β-adrenergic receptors (β-AR), and promotes glycogenolysis to provide energy support [46]. Retinol and its derivatives such as retinoic acid in retinol metabolism participate in the myocardial development process [47], indicating that (−)-IFZ mainly affected the heart development and function of larvae. The rac-IFZ exposure group exhibited significant enrichment in pathways associated with drug metabolism, with xenobiotic metabolism by cytochrome P450 enzymes, particularly involving CYP450 isoforms, serving as the central enzymatic system responsible for the biotransformation of foreign compounds [48]. Cytochrome P450 (CYP450), a heme-containing monooxygenase predominantly localized in the hepatic and extrahepatic endoplasmic reticulum [49], requires both NADPH coenzyme and molecular oxygen for its catalytic activity. This enzyme system primarily mediates the oxidative biotransformation of xenobiotics through various reactions including electron transfer, dehydrogenation, and oxygenation processes [50]. Studies have shown that triazole fungicides mainly inhibit the 14α-demethylation reaction mediated by fungal cytochrome P450, and block ergosterol synthesis, thereby destroying the cell membrane structure and exerting an antibacterial effect. This also showed that IFZ had the typical action characteristics of triazole fungicides.

To further compare the differences in toxic effects between (+)-IFZ and (−)-IFZ, transcriptomic analysis was performed on the DEGs of (+)-IFZ relative to (−)-IFZ, and GSEA analysis was performed on the four signaling pathways of fatty acid degradation, PPAR signaling pathway, adrenergic signaling in cardiomyocytes, and cardiac muscle contraction. The observed upregulation of both PPAR signaling and fatty acid degradation pathways suggested a coordinated activation of lipid metabolic processes, characterized by enhanced fatty acid β-oxidation and subsequent alterations in cellular energy homeostasis [51]. The observed enhancement of adrenergic signaling within cardiomyocytes, along with the potentiation of cardiac muscle contraction pathways, reflects a compensatory physiological response in juvenile fish. Under conditions of oxidative stress, this adaptive mechanism supports the maintenance of adequate organ perfusion through sympathetic nervous system activation and an increase in myocardial contractile force [52,53]. Furthermore, elevated heart rate and enhanced cardiac contractility resulted in greater ATP demand, triggering concurrent activation of fatty acid β-oxidation pathways to meet energy requirements.

While qPCR validation of selected genes can provide additional support for RNA-seq findings, the confirmation of transcriptome-based predictions derives from consistent phenotypic outcomes, such as hepatic developmental defects, compromised cardiac function, and altered expression of key genes, which directly reflect the biological impact of pathway dysregulation.

## 5. Conclusions

To evaluate the toxicity of IFZ enantiomers, zebrafish larvae were treated with rac-IFZ, (+)-IFZ, and (−)-IFZ. Developmental endpoints such as growth, cardiac and hepatic morphology, and organ function were subsequently assessed. The results revealed distinct enantiomer-specific toxicological profiles among the three treatments. Rac-IFZ, (+)-IFZ, and (−)-IFZ all significantly impaired larval growth and development, while (+)-IFZ and (−)-IFZ both significantly affected lipid metabolism and cardiac development and function. Subsequently, comparative transcriptome analysis was used to characterize enantiomer-specific gene expression profiles and identify distinct toxicological pathways among the three treatment groups, thereby elucidating the molecular mechanisms underlying their distinct phenotypic effects. The results revealed that rac-IFZ primarily disrupted drug metabolism pathways, (+)-IFZ preferentially altered lipid homeostasis, and (−)-IFZ exhibited cardiotoxicity, primarily affecting cardiac development and function. These findings suggest that chiral configuration significantly influences the toxicological targets and outcomes of IFZ in developing zebrafish.

## Figures and Tables

**Table 1:** Effects of Ipfentrifluconazole exposure on the biochemical indicators of zebrafish larvae.

Biochemical Indicators	Ipfentrifluconazole			
	CON	Rac-Ipfentrifluconazole	(+)−Ipfentriflucona-zole	(−)−Ipfentriflucona-zole
TG (mmol/gprot)	0.016 ± 0.001	0.023 ± 0.001 **	0.028 ± 0.001 **	0.024 ± 0.000 **
TC (mmol/gprot)	0.083 ± 0.002	0.087 ± 0.001	0.089 ± 0.002	0.098 ± 0.003 **
Gluscode (mmol/gprot)	0.086 ± 0.003	0.084 ± 0.002	0.091 ± 0.003	0.099 ± 0.003 *
Pyruvate (mmol/gprot)	0.027 ± 0.003	0.026 ± 0.002	0.025 ± 0.002	0.024 ± 0.001

Full name of biochemical indicators: TG, total triglycerides; TC, total cholesterol. The presented values are the mean ± SEM (*n* = 6), * *p* < 0.05; ** *p* < 0.01 versus control group.

## Data Availability

Data are available by contacting the authors.
